# Profiling of the small RNA populations in human testicular germ cell tumors shows global loss of piRNAs

**DOI:** 10.1186/s12943-015-0411-4

**Published:** 2015-08-12

**Authors:** TB Rounge, K Furu, RI Skotheim, TB Haugen, T Grotmol, E Enerly

**Affiliations:** Cancer Registry of Norway, Oslo, Norway; Department of Molecular Oncology, Institute for Cancer Research, Norwegian Radium Hospital, Oslo University Hospital, Oslo, Norway; Centre for Cancer Biomedicine and Institute of Informatics, University of Oslo, Oslo, Norway; Faculty of Health Sciences, Oslo and Akershus University College of Applied Sciences, Oslo, Norway

**Keywords:** Small non-coding RNA, piRNA, miRNA, tRNA-derived small RNA, Testicular cancer

## Abstract

**Background:**

Small non-coding RNAs play essential roles in gene regulation, however, the interplay between RNA groups, their expression levels and deregulations in tumorigenesis requires additional exploration. In particular, a comprehensive analysis of microRNA (miRNA), PIWI-interacting RNAs (piRNAs), and tRNA-derived small RNAs in human testis and testicular germ cell tumor (TGCT) is lacking.

**Results:**

We performed small RNA sequencing on 22 human TGCT samples from 5 histological subtypes, 3 carcinoma *in situ*, and 12 normal testis samples. miRNA was the most common group among the sequences 18–24 nt in length and showed histology-specific expression. In normal samples, most sequences 25–31 nucleotides in length displayed piRNA characteristics, whereas a large proportion of the sequences 32–36 nt length was derived from tRNAs. Expression analyses of the piRNA population demonstrated global loss in all TGCT subtypes compared to normal testis. In addition, three 5′ small tRNA fragments and 23 miRNAs showed significant (*p* < 10^−6^) differential expression in cancer vs normal samples.

**Conclusions:**

We have documented significant changes in the small RNA populations in normal adult testicular tissue and TGCT samples. Although components of the same pathways might be involved in miRNA, piRNA and tRNA-derived small RNA biogenesis, our results showed that the response to the carcinogenic process differs between these pathways, suggesting independent regulation of their biogenesis. Overall, the small RNA deregulation in TGCT provides new insight into the small RNA interplay.

**Electronic supplementary material:**

The online version of this article (doi:10.1186/s12943-015-0411-4) contains supplementary material, which is available to authorized users.

## Background

In recent years, it has become increasingly evident that many non-protein-coding regions of the genome are transcribed [1], giving rise to non-coding RNAs (ncRNA) that play crucial roles in normal biological processes and human diseases [2]. Within the diverse group of ncRNA, small non-coding RNAs (sncRNAs) have emerged as potential important regulators of gene expression [3]. These RNA molecules are highly complex in terms of structural diversity and function. They are typically 19–35 nucleotides (nt) in length, interact with Argonaute family proteins [4–7], and include microRNAs (miRNAs), and PIWI-interacting RNAs (piRNAs). Precise control of miRNA expression is crucial for keeping cells in normal physiological states, and dysregulation of miRNAs may lead to oncogenesis [8, 9]. The miRNA pathway is thought to be important for spermatogenesis [10] and several miRNAs have been found to be exclusively or preferentially expressed in the testis [11]. Moreover, the miRNAs miR-371-373, miR-302 and miR-146 have previously been shown to display a TGCT-specific expression pattern [12–14], indicating a potential role for miRNAs in TGCT pathogenesis.

Unlike miRNA expression, piRNAs are mainly restricted to the male germline [5, 15–17], but have recently also been found in small amounts in somatic tissues, including cancer [18–21]. It has been reported that piRNAs tend to be generated in a clustered fashion from specific loci in the genome [5, 16], and that they are mainly transcribed from regions containing transposons and other repetitive elements [2, 22]. They are generated from long primary transcripts that are processed by unknown endonucleases into mature piRNAs [22, 23]. Their roles in humans are still unclear, but studies in model organisms suggest a role in transposable element regulation and DNA methylation [23–28]. It is thought that they form piRISC complexes with PIWI proteins and target mRNAs for cleavage or translational repression by binding to complementary sequences in target mRNAs [29, 30].

Other classes of sncRNAs have also been identified, including tRNA-derived small RNAs, a class of small RNA generated by specific endonucleases from mature tRNAs in response to certain conditions, such as oxidative stress, heat shock, or nutrient deprivation [31–33]. Previous studies have shown that tRNA-derived small RNAs can be divided into two main groups; small tRNA fragments (tRFs) (~20 nt in length) and tRNA halves (~30 nt in length) [32]. Their mode of action is still unclear, but they may influence cell proliferation, probably through translational arrest [32, 33]. Yamasaki and coworkers showed that stress induction in mammalian cells leads to formation of tRNA halves that inhibit translation [33]. This inhibitory effect is specific for sequences derived from the 5′ end of mature tRNAs [34]. Recently, Keam and co-workers showed that HIWI2, a human PIWI homolog, binds 5′ tRNA-derived small RNAs in a human breast cancer cell line, indicating crosstalk between the piRNA and tRNA pathways [20]. Similar tRNA-derived small RNAs have also been detected in all stages of mouse spermatogenesis [35].

Small ncRNA may play an important role in TGCT. TGCT develops from carcinoma *in situ* (CIS; alias intratubular germ cell neoplasia) lesions that may arise *in utero* from primordial germ cells (PGCs) [36] and is the most common malignancy in young men in most western countries [37, 38]. The etiology of TGCT is largely unknown, although genetic components and conditions during pregnancy play a role [39–41]. In CIS cells, the demethylation machinery maintains the genome in a generally demethylated state, much like in undifferentiated PGCs [42], indicating an epigenetic component in the development of testicular neoplasia. Recently, downregulation of the PIWI-piRNA pathway in human TGCTs was shown by Ferreira and coworkers [43]. Downregulation was associated with loss of LINE-1 methylation. Combined, these findings indicate that epigenetic disruption is a hallmark for the development of testicular tumors, and that it is affected by the sncRNA expression.

Despite the emerging biological significance of sncRNAs, most studies thus far have been conducted in model organisms. Therefore, the abundance, diversity, origin, and function of human sncRNAs are still relatively unknown. Although several studies have been performed on the role of miRNAs in spermatogenesis and TGCT, little is known about the presence and molecular function of other classes of human testicular sncRNAs. This exploratory study elucidates the presence and expression levels of small RNA populations in normal testicular tissue and TGCT histological subtypes. We analyzed the distribution of sncRNAs across the human genome by small RNA sequencing on RNA isolated from a total of 37 human samples.

## Results

### The small RNA populations differ between normal and TGCT tissue

The resulting small RNA dataset encompasses samples from all major histological TGCT subtypes and relevant cell lines, and yielded almost 379 million sequence reads (Table 1). The lowest average sequence count came from the EC-cell lines, and the highest from CIS samples.

**Table 1:** Data processing metrics for small RNA reads

Histology	No of samples	Sequences	Aligned	Unique aligned	Assigned to miRNA^d^	Assigned to small RNA contigs
Normal^a^	12	137,821,767	107,214,046 (77.8 %)	69,211,744 (50.2 %)	44,584,258	361,269,586
Carcinoma *in Situ* ^b^	3	46,285,700	36,839,326 (79.6 %)	27,608,395 (59.6 %)	24,977,605	55,763,124
Embryonal Carcinoma cell lines^c^	6	24,651,693	14,504,501 (58.8 %)	12,270,382 (49.8 %)	4,076,105	12,679,068
Embryonal Carcinoma	5	60,049,727	45,515,442 (75.8 %)	22,271,004 (37.1 %)	14,412,704	21,9138,834
Yolk Sac Tumor	4	33,808,192	25,482,718 (75.4 %)	14,831,132 (43.9 %)	15,221,965	80,580,426
Seminoma	3	24,687,396	18,385,722 (74.5 %)	12,754,266 (51.7 %)	5,327,777	52,606,953
Teratoma	3	43,576,658	35,638,201 (81.8 %)	21,503,877 (49.3 %)	30,109,084	76,746,231
Chorio-carcinoma	1	80,68,838	6,171,431 (76.5 %)	4,171,369 (51.7 %)	4,545,330	8,955,051
TOTAL	37	378,949,971	289,751,387 (76.5 %)	184,622,169 (48.7 %)	143,254,828	867,739,273

Raw sequence data from the small RNA sequencing dataset. Percentage >100 % is due to multiple hits in the sequence mapping. ^a^The normal samples are from 11 healthy men and 1 biopsy from histological normal tissue adjacent to a tumor in a TGCT patient. ^b^ The cell line samples consists of cells from the embryonal carcinoma lines NT2 and 2102Ep. ^c^ Evaluation of cryosections of the carcinoma *in situ* samples estimated presence of CIS cells in 10 %, 50 %, and 100 % of the seminiferous tubuli. ^d^ As annotated by miRBase

**Table 2:** Top 10 differentially expressed piRNA clusters (A), tRNA-derived small RNAs (B) and miRNAs (C)

**A**	**Top 10 differentially expressed small RNA clusters**		
	Genomic location	Log2 Fold Change	*P*-value (adjusted)
1	chr15:21,925,462-21,930,432	−6.18	2.62e-29
2	chr15:20,841,664-20,850,393	−6.18	1.31e-27
3	chr15:23,510,099-23,514,854	−6.25	1.46e-26
4	chr18:55,837,529-55,838,674	inf	1.46e-26
5	chr1:2,237,998-2,241,778	−6.55	1.88e-26
6	chr15:23,386,898-23,404,357	−6.06	1.88e-26
7	chr15:28,583,287-28,593,483	−5.93	1.88e-26
8	chr4:190,801,280-190,804,618	−6.21	1.88e-26
9	chr15:20,723,798-20,737,584	−5.94	3.27e-26
10	chr18:14,460,205-14,464,963	−6.30	3.27e-26
**B**	**Differentially expressed tRNA-derived small RNAs**		
	tRNA	Log2 Fold Change	*P*-value (adjusted)
1	Glu-GAG (chr13, 45492060)	−2.51	2.50e-09
2	Glu-GAG (chr15, 26327380)	−2.63	2.50e-09
3	Asp-GAY (chrX, 18996334)	−3.49	3.40e-06
**C**	**Top 10 differentially expressed miRNAs**		
	miRNA	Log2 Fold Change	*p*-value (adjusted)
1	hsa-miR-302d-3p	11.25	1.95e-13
2	hsa-miR-302a-3p	11.65	1.23e-12
3	hsa-mir-302b	11.38	1.23e-12
4	hsa-mir-302a	11.18	7.26e-12
5	hsa-miR-367-3p	10.20	1.48e-11
6	hsa-miR-371a-5p	9.29	2.09e-11
7	hsa-miR-371b-3p	9.29	2.09e-11
8	hsa-miR-302c-5p	Inf	3.45e-11
9	hsa-miR-302d-5p	Inf	1.38e-10
10	hsa-miR-302c-3p	11.32	1.38e-10

After sequence trimming, 76.5 % of the sequences mapped to the reference genome, of which 63.7 % mapped uniquely. In consistence with the piRNA signature observed in previous studies [5, 16, 25], a proportion of the putative piRNA sequences in each sample group mapped to several loci in the genome.

**Fig. 1:**
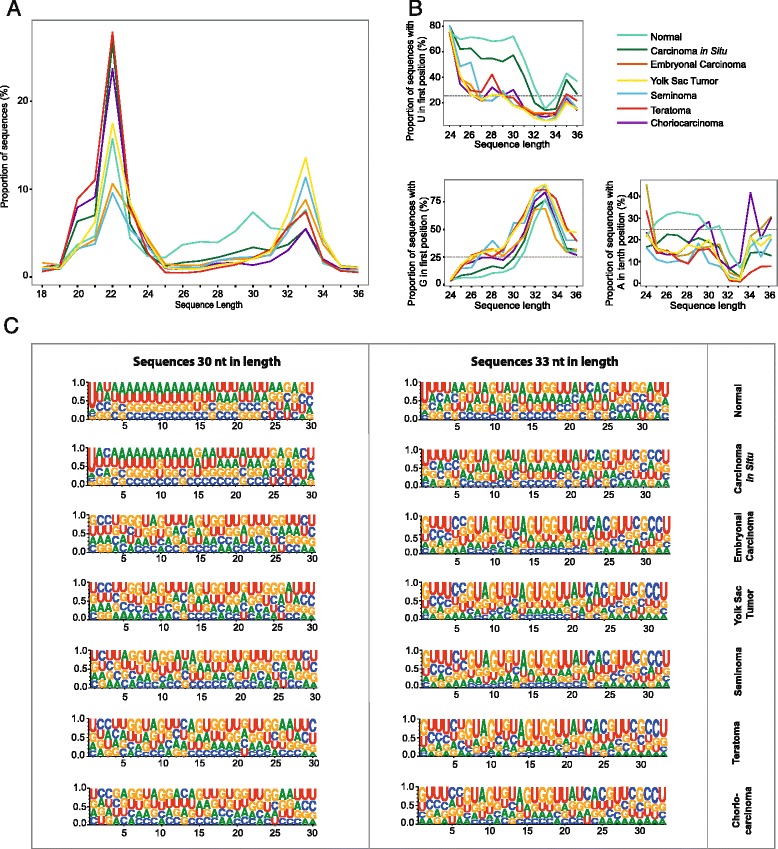
Small RNA length distribution and base compositions in normal and TGCT samples. **a** Small RNA sequence length distribution in each sample group (sequences 18–36 nt in length) based on normalized counts in each sample. **b** The percentage of reads aligning to the human genome with U in first position, G in first position, and A in 10^th^ position for unique small RNA sequences 24–36 nt in length in each sample group. The horizontal line at 25 % in the plots represents the expected base proportion under random distribution. **C** Web logos for unique, trimmed sequences 30 and 33 nt in length showing the base composition in each nucleotide position. The X-axis represents the nucleotide position relative to the 5′ end, whereas the Y-axis represents the entropy score for the base bias

The small RNA length distribution shows abundant sequences in the size range 19 to 36 nt (Fig. 1a). The length distribution for the normal samples shows three peaks centered at 22, 30 and 33 nt. The length of the sequences in the two former peaks is consistent with the presence of miRNAs and piRNAs, respectively [17, 44]. The piRNA peak is lower in the TGCT samples compared to the normal samples, demonstrating a difference in the small RNA populations in normal and TGCT samples. Sequence conservation at each position differs between TGCT, CIS and normal samples. Consistent with the presence of piRNAs, nearly 70 % of the sequences from normal samples 24–30 nt in length contain uridine in the 5′ position (5′U; Fig. 1b-c), and we also observe a slightly elevated proportion of sequences containing adenine in the 10^th^ position (10A). The proportion of 5′U and 10A show a strong decrease from 30 to 33 nt sequence length. The CIS subtype has a base composition pattern that resembles that of the normal samples, whereas the TGCT samples display a random distribution of the nucleotides in position 1 and 10 (probability for each base close to 25 %) (Fig. 1b-c). Additionally, sequences 32–34 nt in length in all sample groups show a significant enrichment for 5′G, corresponding to the decrease in sequences containing 5′U (Fig. 1c). The cancer sequences >30 nt in length contain a large proportion of similar sequences (data not shown), resulting in the appearance of a AGUGGUU motif in positions 14–20 (Fig. 1c).

**Fig. 2:**
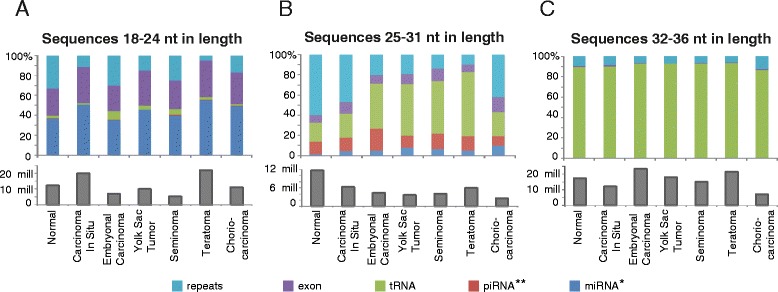
Relative distribution of small RNA sequences in three classes according to annotation. The sequences are divided into three categories based on the peaks in the size distribution, corresponding to miRNAs, piRNAs and longer sequences of previously unknown origin. The histograms indicate the average sequence count per sample in each group. * As annotated by miRBase, ** As annotated by piRNABank

The sequences were assigned to different classes based on annotations of the mapped region and the sequence lengths corresponding to the three peaks observed in the length distribution plot. The aligned sequences make up between 58.8 – 81.1 % of all sequences (Table 1). A large proportion of the sequences 18–24 nt were annotated as miRNAs (Fig. 2a), whereas around 30 % of the sequences were derived from exons. The distribution of annotated sequences was similar for all histological subgroups. Sequences 25–31 nt in length, however, vary with sample groups (Fig. 2b and Additional file 1). In particular, 60.0 % of the normal sequences were derived from repeats, whereas repeat-associated sequences constitute 9.6 – 41.8 % of the sequences in the TGCT samples.

The absolute difference in sequence counts between normal and TGCT samples in this size range are mostly due to low abundance of repeat-associated sequences in the TGCT samples (on average 10.5 and 4.2 mill sequences, respectively, not including the CIS samples) (Additional file 1). This reduction is in accordance with loss of the 30 nt peak observed in the TGCT sequence length distribution plot (Fig. 1a). In addition, a large proportion of the TGCT sequences 25–31 nt in length are derived from tRNAs. This tRNA enrichment was even more pronounced in sequences 32–36 nt in length, where ~90 % of the sequences in all sample groups were tRNA-derived (Fig. 2c). The CIS samples have a sequence annotation distribution intermediate between normal testis and TGCT samples.

### The piRNA biogenesis pathway is globally downregulated in TGCT samples

Mature piRNAs originate from processing of longer RNA precursors [45]. We merged direction-specific overlapping small RNA sequences into contigs allowing gaps up to 100 bp, mimicking piRNA precursors. Sequences 24 to 35 nt in length were included to be able to study both putative piRNAs and tRNA-derived sequences. A total of 184,344 small RNA contigs were generated, with a mean and median length of 516 and 188 bases, respectively.

**Fig. 3:**
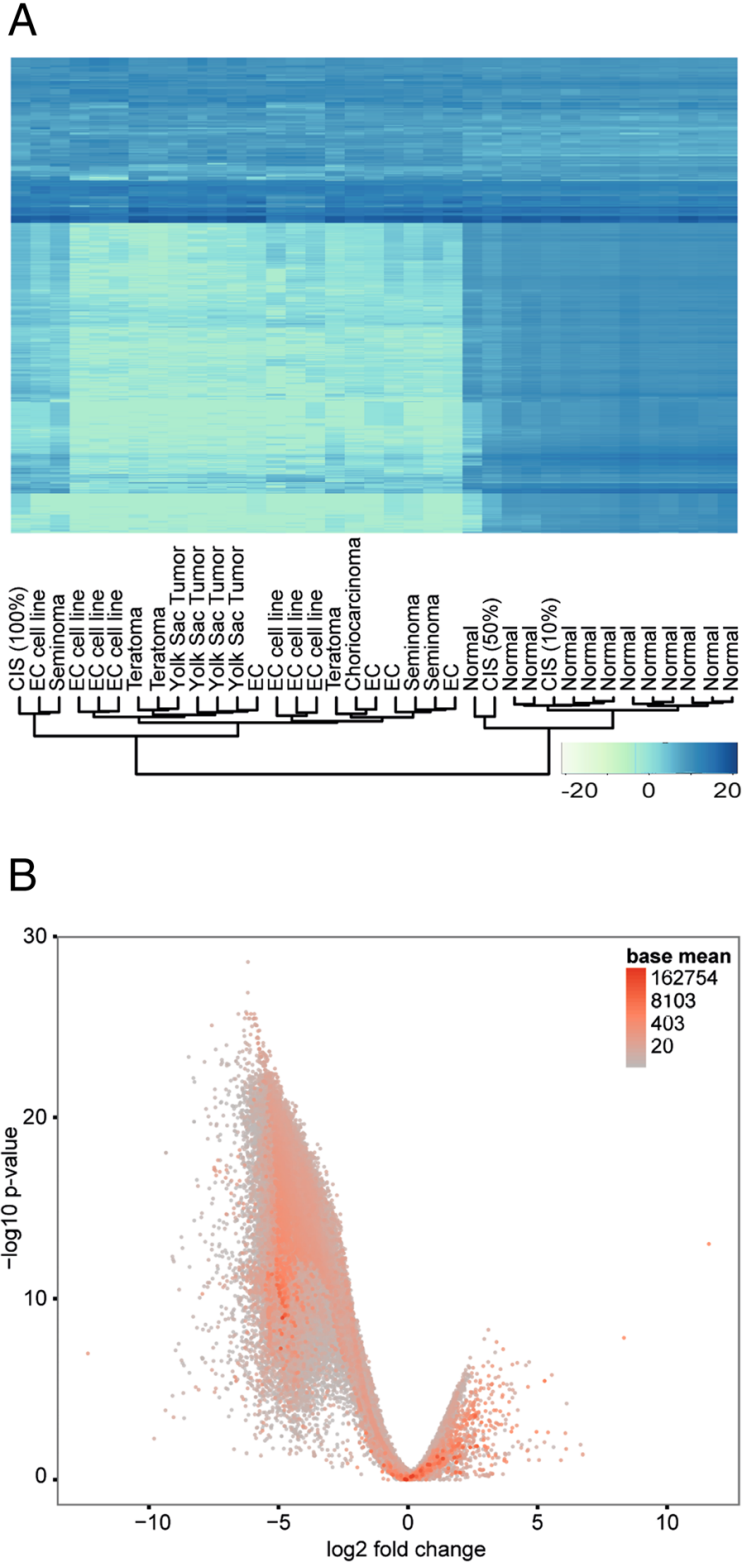
Small RNA contig expression in normal and TGCT samples. **a** Heatmap showing the expression data of the 2000 most highly expressed piRNAs (variance stabilization transformed data). The dendrogram (bottom) indicates clustering according to expression profiles. Heatmap colors represent relative small RNA contig expression as indicated in the color key (bottom right). **b** Volcano plot indicating the relationship between the Log2 fold change and p-values (Benjamini-Hochberg adjusted) for all small RNA contigs when comparing TGCT with normal samples. The CIS samples are included in the TGCT group. Color coding indicates the expression level as a function of normalized counts (BaseMean)

Differential expression analysis of contigs from normal testis and TGCT samples (including CIS samples) revealed a genome-wide loss of small RNA contigs in the TGCT samples (Fig. 3a). This loss leads to the asymmetric, left-skewed shape of the volcano-plot observed in Fig. 3b, revealing an average log2 fold change of −5. The most differentially expressed small RNA contigs are listed in Table 2A, all of which are down-regulated in TGCT. The 5′U preference in the sequences 25–31 nt in length indicate that these sequences are mainly piRNAs. piRNABank consists of 667,666 piRNA sequences and 114 high density regions, called piRNA clusters [46]. All piRNABank clusters and 20 % (131,176) of the sequences overlap with the small RNA contigs in our dataset. 47 % (86,240) of the 184,344 small RNA contigs overlap with piRNABank sequences. Differential expression analyses of sequences overlapping with piRNA bank sequences show the same expression pattern as the small RNA contigs, although with lower counts (Additional file 5). A table indicating the top 10 differentially expressed piRNAs when including sequences overlapping with piRNABank only, is given in Additional file 6. Hierarchical clustering showed that normal testis samples and TGCT samples clustered separately, but with no sub-clustering related to TGCT histological subtypes (Fig. 3a).

Despite the global downregulation of small RNA contigs in TGCT, a subset of the contigs was expressed in all samples. Further analysis showed that these sequences are mainly tRNA-derived. We therefore identified all sequences overlapping with known tRNA genes and performed differential expression analyses for tRNA halves and tRFs, based on sequence length. No sequences corresponding to tRNA halves showed significant expression between TGCT and normal testis (*p* > 10^−4^; data not shown). Differential expression analyses of the tRNA-associated sequences ~20 nt in length (tRFs) indicated a significant difference in the sequences derived from three tRNA genes, all downregulated in TGCT (*p* < 10^−6^, Table 2B).

Hierarchical clustering based on data from tRNA halves and tRFs did not group the samples according to histology (Additional file 2A-B). However, sequences derived from tRNAs of the same isotype cluster together, as apparent by the horizontal groups in the heatmaps (Additional file 2A-B). This can also be seen in the corresponding volcano-plots, which display a similar distribution of the sequences in the TGCT and normal testis samples (Additional file 2C-D). Most tRNA isotypes are represented, but tRNAs encoding the amino acids Val, Gly, Glu and Lys are overrepresented in the 100 most highly expressed sequences in both groups. The expression of corresponding tRNA halves and tRFs correlate (log scale), however, a few tRNAs (tRNA-Val-GTG/GTY, tRNA-Gly-GGY/GGG) have a higher abundance in tRFs compared to tRNA halves (Additional file 3A-B). The tRNA-derived sequences are mostly derived from the 5′ end of mature tRNAs (representative example of reads derived from a tRNA gene is given in Additional file 3C).

### Expression analysis of miRNAs shows upregulation of the miR-302 and miR-371-373 clusters

To assess the miRNA population in our samples, sequences with lengths corresponding to miRNAs (17–23 nt in length) were analyzed. A total of 23 miRNAs show differential expression between normal testis and TGCT samples (p < 10^−6^). The top 10 most differentially expressed miRNAs are listed in Table 2C, and an extended list containing all 23 significant miRNAs is given in Additional file 4.

**Fig. 4:**
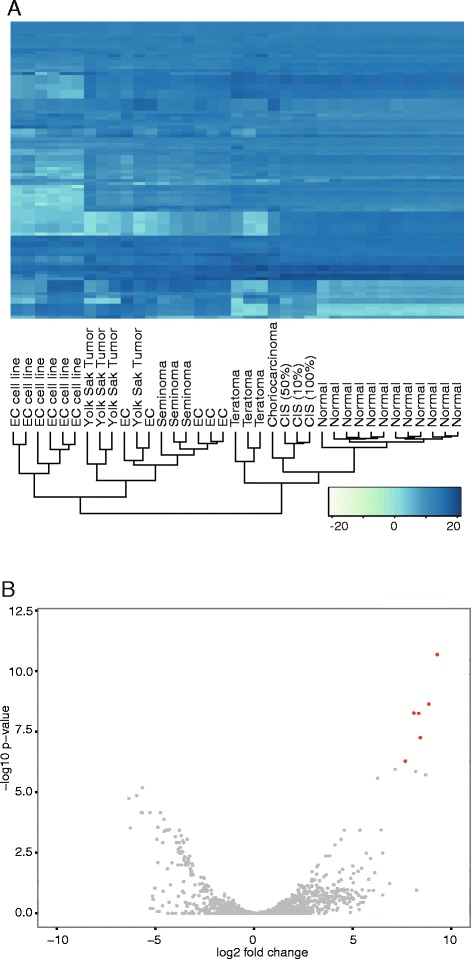
miRNA expression in normal and TGCT samples. **a** Heatmap showing the expression data of the 100 most highly expressed miRNAs (variance stabilization transformed data). The dendrogram (bottom) indicates clustering according to expression profiles. Heatmap colors represent relative miRNA expression as indicated in the color key (bottom right). **b** Volcano plot indicating the relationship between the Log2 fold change and p-values (Benjamini-Hochberg adjusted) for all miRNAs when comparing TGCT with normal samples. The CIS samples are included in the TGCT group. Red color coding indicates a significance level of *p* < 10^−8^ on a –log10 scale and log2 fold change above 5/below −5

The miRNA expression profiles reflect TGCT histological subtypes. Hierarchical clustering generated a dendrogram aligning with the experimental factors (cases and controls) and TGCT histological subtypes (Fig. 4a). The CIS samples cluster closely together with the normal samples, further verifying our observations that CIS represents an intermediate stage between normal and malignant testicular tissue.

In contrast to the results on small RNA contig expression, the volcano plot for the miRNA data generates a symmetrical pattern, indicating that some miRNAs are downregulated, whereas others are upregulated in TGCT (Fig. 4b). The most significantly differentially expressed miRNAs were from the miRNA clusters miR-302/367 and miR-371-373, seen in the upper right corner of the plot.

**Fig. 5:**
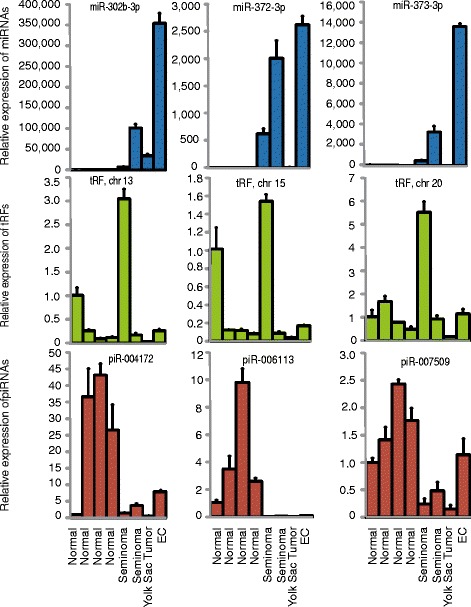
qPCR validation. Three small non-coding RNA sequences in each of the three categories miRNA (blue), piRNA (red) and tRF (green) were selected and their relative expression in 8 samples (4 normal, 4 cancer) was analyzed using qPCR. The first normal sample is used as reference (RQ = 1).

We selected three miRNAs, piRNAs and tRFs for validation using qPCR. The validation shows corresponding up/downregulation to the small RNA sequencing (Fig. 5), although intra-group variation is observed.

## Discussion

During the last decade, several novel classes of sncRNAs have been identified. These molecules are highly complex, and their biogenesis, molecular function and regulation are still largely unknown [47]. With this study we provide, to our knowledge, the first comprehensive characterization of the small RNA sequences in different histological subtypes of TGCT, and testicular carcinoma *in situ*, as well as in normal testis samples. Overall, we show that the human testis is highly abundant in miRNAs, piRNAs and tRNA-derived small RNAs. More specifically, the small RNA population in our dataset showed characteristics of canonical piRNAs, with an overall genomic distribution closely resembling that of a previous study [5], as well as a large overlap with piRNABank [46]. In addition, we confirm the findings from other studies showing that human piRNAs, like piRNAs in other organisms, have a strong preference for 5′ U [25, 48–50]. Together, these results strongly imply that a large proportion of the small RNAs, are indeed piRNAs. In the normal samples, we also observed a slightly elevated proportion of 10A in sequences 24–30 nt in length, indicating that some piRNAs may be generated through the ping-pong mechanism [25, 48, 49].

Most piRNAs are globally downregulated or completely lost in all TGCT histological subtypes. This is not surprising, as tumor tissue has fewer differentiated germ cells, the main producer of piRNAs [5, 15–17] compared to normal testis. Global downregulation of piRNAs in TGCT is supported by the findings of Ferreira and co-workers, describing diminished levels of PIWI proteins and piRNAs both in TGCT primary tumors and cultured transformed cells [43]. Among the TGCT samples, we found 1) no histology-specific piRNA profiles, 2) no enrichment for 5′U and 10A, and 3) a lower read count for sequences 24–30 nt in length. Combined, these data indicate a lack of sequences generated through the piRNA pathway in TGCT. Some piRNAs are, however, detected in the cancer samples, shown by both the small RNA sequencing and qPCR validation, probably originating from a few normal cells within the tumors/biopsies.

TGCT develops from CIS lesions that may arise *in utero* in PGCs or gonocytes [36]. Although PGCs or gonocytes are not present in normal testis, we have considered CIS to represent a transitional state between normal testis tissue and TGCT, evident by the intermediate levels of 5′ U/10A enrichments observed in CIS samples (Fig.1b). Only a proportion of the tubular cells will be affected in CIS (5-10 % abnormal cells [51] in a 100 % CIS sample), generating a mix of normal spermatogenic and neoplastic cells. Almstrup and co-workers have previously determined the three CIS samples included in our study to contain 10 %, 50 %, and 100 % affected seminiferous tubuli, respectively [51]. The samples do not cluster together in the piRNA dendrogram (Fig. 3a), probably due to the differences in the amount of neoplastic cells within the samples. Interestingly, the sample containing the highest amount of affected tubules cluster together with an embryonic carcinoma (EC) sample, whereas the sample containing the lowest amount of affected tubules clusters together with the healthy controls.

The high abundance of tRNA-derived RNAs 32–36 nt in length, representing tRNA halves, is in accordance with other studies [20, 35]. Differential expression analyses of tRNA-derived sequences showed that despite their high abundance, no tRNA halves are differentially expressed between TGCT and normal tissue. This indicates that the population of tRNA halves is not affected by tumorigenesis. Most tRNA halves were found to be derived from the 5′ end of mature tRNA [20, 35], suggesting that they are not degradation products, but rather processed tRNAs. The overrepresentation of only a few tRNAs, is similar to the results in other studies [20, 35].

In addition to tRNA halves, we identified tRNA-derived sequences ~20 nt in length, corresponding to tRNA Fragments (tRFs) [47]. These tRFs may be produced from the 5′- or 3′-end of mature tRNAs by Dicer, and associate with AGO proteins to participate in various processes of transcriptional and post-transcriptional regulation [52–54]. Not much is known about the role of tRNA-derived small RNAs in cancer. It it has been reported that the levels of mature tRNAs are generally elevated in cancer [55], whereas our results indicate that the levels of tRNA halves and tRFs are relatively stable in TGCT. The findings by Keam et al., showing that tRNA-derived small RNAs bind to HIWI2 [20], indicate crosstalk between sncRNA pathways and may indicate that the sncRNA classes are more overlapping in terms of function and biogenesis than previously thought. Our results, however, show that the response to the carcinogenic process differs between these pathways, suggesting independent regulation of their biogenesis. More research is needed to elucidate the potential targets of tRNA-derived small RNAs and their role in cancer, including in TGCT.

Whereas piRNAs are almost exclusively found in the male gonad [5, 16, 17], miRNAs are expressed in most cell types. The miRNA population varies, however between tissues. We speculate that the observed differences in miRNA profiles are driven by differences in the cellular origins of the TGCT subtypes, whereas piRNAs are lost in the carcinogenic cells due to the spermatogenesis-specific expression of this pathway. This lack of piRNA defense in CIS and TGCT cells may be a factor in testicular carcinogenesis, causing reduced ability to prevent chromatin instability. Diminished piRNA expression and hypomethylation events at LINE-1 loci in TGCT are supported by the findings of Ferreira et al. [43] and Ushida et al. [56].

Our results confirm previous findings indicating that miRNAs have a relevant role during testicular carcinogenesis, since overexpression of the miR-371-373, miR-302 and miR-367-3p clusters was noted in malignant TGCT tissue [12, 57, 58]. These miRNAs are implicated in TGCT development [13], maintenance of pluripotency [59], and in cisplatin sensitivity [60]. A study using a genetic screen of primary human cells supported this observation and found that both miR-372-373 and miR-302 may act as TGCT oncogenes through inhibition of target genes such as Large Tumor Suppressor homolog 2 (LATS2) [12]. These miRNAs are all highly upregulated in TGCT, indicating a role as oncogenes in TGCT tumorigenesis. Also among the significantly differentially expressed miRNAs are miR-200c and miR-141, both belonging to the miR-200c/141 cluster, which has been found to act as inhibitors of the epithelial-to-mesenchymal transition, tumor cell invasion, and metastasis in several cancers [61–63]. Counter indicative of their role as tumor suppressors, these miRNAs were also found to be upregulated in TGCT. However, there are some tumor types in which upregulation of the miR-200c/141 cluster has been observed, including ovarian carcinoma and endometrial carcinoma [64, 65].

A limitation of the present study is the possible presence of non-piRNAs among the 25–32 nt long sequences. Although we enrich for phosphorylated small RNAs with a 5′-OH group in the library preparations, degraded RNAs and other non-phosphorylated RNAs may be co-sequenced. Compared to using PIWI protein immunoprecipitation [5, 17], our method gives a broader range of small RNAs and a somewhat higher degree of non-targeted sequences. The strong preference for 5′U and the large overlap with piRNABank sequences, indicate that most of our sequences 25–32 nt in length are indeed piRNAs. In addition, differential expression analysis of only the sequences overlapping with piRNABank showed a similar expression pattern, indicating a true loss of the piRNA pathway in TGCT.

Another possible bias in the small RNA expression measurements is differences in RNA extraction protocols [66]. Most normal samples were extracted using a phenol-free protocol, while the TGCT sample RNA extraction included a phenol step. To investigate this weakness in our design, the small RNA profiles of the available phenol-free (*n* = 9) and phenol extraction (n = 3) of the normal samples were compared. No significant differential expression was found between samples prepared with the two extraction methods. Regardless, a replication study is needed to confirm potential biomarkers.

There is a need for more sensitive biomarkers for TGCT detection and surveillance [67]. Several of the miR-371-373 and miR-302/367 cluster members have shown a sufficiently strong association with TGCT [12, 57, 58] to serve as biomarkers of TGCT. Accordingly, serum levels of these miRNAs have been investigated and were found to be significantly higher in TGCT patients than in healthy controls, as well as display decreasing levels upon treatment [68–70]. Among the miRNAs members from these clusters, miR-371a-3p and miR-367-3p have been considered most promising [69, 71]. We have confirmed the association between TGCT and high expression of the miR-371-373 and miR-302/367 clusters. Despite our findings that differentially expressed piRNAs and tRNA-derived small RNAs are mostly downregulated in TGCT, they may have potential as biological markers. More research is needed to determine the role of these sequences in TGCT carcinogenesis and their potential clinical use.

## Conclusions

We have documented significant changes in the small RNA populations in normal adult testicular tissue and TGCT samples. Most notable, our results indicate loss of the piRNA biogenesis pathway in TGCT, independent of histological subtype, and presence of large amounts of tRNA-derived small RNAs in both normal testicular tissue and in TGCT samples. Although crosstalk between small RNA pathways has been suggested, our results indicate independent regulation of miRNA, piRNA and tRNA-derived sequences in TGCT. These results may contribute to increased understanding of the functional roles of sncRNAs and their role in human spermatogenesis. In addition, they indicate that sncRNAs play an important role in TGCT development and progression, and that they may be promising candidate diagnostic markers for TGCT.

## Methods

### Sample collection and handling

Testicular tissue samples were selected from two sources; i) a series of primary TGCTs previously analyzed by Skotheim and co-workers [72], and ii) a series of normal testicular tissue samples obtained from adult organ transplant donors, in total 37 samples (Table 2). Rough estimate of cryosections from the three CIS samples has previously indicated the proportion of affected tubules to be 10 %, 50 %, and 100 % [51]. The project has been approved by the National Committee for Medical and Health Research Ethics (S-05368 and S-07453b).

### Small RNA library preparation and sequencing

Total RNA extracts from all samples (isolated using phenol extraction or RNeasy (Qiagen, USA)) were used for library preparation and small RNA sequencing. Small RNA sequencing libraries were generated using the Illumina TruSeq Small RNA Sample Preparation protocol (Illumina Inc., San Diego, CA, USA). One microgram total RNA was ligated to 3′- and 5′-RNA adapters, and ligation products were reverse transcribed to generate cDNA libraries for each sample. Products were PCR amplified, where unique index sequences were incorporated, before pooling of the libraries and gel purification. The samples were size selected on a 6 % PAGE gel, cutting from the upper side of the 160 bp marker to the lower side of the 140 bp marker, ensuring inclusion of small RNAs in the size rage 18–36 nucleotides. Small RNA libraries were sequenced single read 50 bp using an Illumina HiSeq2500 sequencer (Illumina Inc., San Diego, CA, USA). Library construction and sequencing was performed at the Oslo University Hospital Genomics Core Facility (http://oslo.genomics.no),

### RNA-seq data processing and expression analysis

Adaptors, short reads (<17 nt) and low quality bases were removed using Nesoni clip v0.128 (http://www.vicbioinformatics.com/software.nesoni.shtml). Small RNA length distribution was calculated from the trimmed fastq output files, and the nucleotide distribution in position 1 and 10 were calculated (custom scripts, available upon request). Unique sequences from each histological subtype were used as input to visualize the probability of each nucleotide in a weblogo (v3.3) for sequences 30 and 33 nt in length [73]. Sequences longer than 17 nt were aligned to the human reference genome (GRCh37) using Novoalign (V3.02.00) with settings recommended for miRNA alignments allowing maximum one mismatch (parameters: −r All 100 -e 100 -m -l 16 -t 30) (www.novocraft.com).

Sequences overlapping with features such as repeatmasker (includes tRNAs), exon and RefSeq tracks from the UCSC table browser (http://genome.ucsc.edu), piRNA clusters in piRNA bank [46], and mature miRNAs in miRBase [74] were investigated using intersectBed from BEDtools [75]. The piRNA Bank clusters were mainly identified using a sliding window method from sequences associated with PIWI proteins [5]. We assumed that a set of overlapping sequences mapping to a genomic region is generated from the same primary transcript [22, 23], thus all sequences with a length between 24 and 36 nt matching the human genome were merged to small RNA contigs using the BEDTools option bedtools merge [75]. Gaps up to 100 bp between sequences were allowed and contigs built from less than 100 sequences in total were discarded. These regions were called small RNA contigs and were used as units for measuring differential expression. Sequences that mapped to the small RNA contigs, miRNAs in miRBase v20 [74], and tRNAs were quantified using FeatureCounts from the subread package v1.4.3 [76]. To avoid disregarding a large proportion of piRNAs mapping to repeat sequences, piRNAs mapping to multiple loci in the genome were included in the analysis.

Differential expression between cancer and normal samples was calculated using counts of small RNA data from FeatureCounts and the DESeq package v1.16.0 for the statistical environment R [77]. For miRNA differential expression, dispersion was estimated using a pooled method, maximum sharing mode and parametric fit type, while for the much larger small RNA contig dataset a pooled method, maximum sharing mode and a local fit type was used. Heatmaps were produces with heatmap.2 from the qplot R package using variance stabilized count data and Euclidean measure to obtain distance matrix and complete agglomeration method for clustering. Differences between the mean of normalized counts for miRNAs and small RNA contigs from cancer and normal samples were calculated using the nbinomTest.

### qPCR validation

RNA from the same extraction used for sequencing was also used for quantitative PCR validation. A total of 8 samples (4 normal, 2 seminomas, 1 EC, and 1 YST) were selected and cDNA synthesis was performed on 0.2 μg totalRNA using miScript II RT Kit (Qiagen, USA) according to the manufacturer’s instructions. The qPCR reactions were carried out on a Stratagene Mx3000p instrument using 1 ng cDNA, miScript SYBR Green PCR kit (Qiagen, USA) and miScript primer assays (Qiagen, USA). Sequences found to have a differential expression (*p* < 10^−6^) in the sequencing dataset were selected for validation. Primers for miRNA detection were ordered from the Qiagen pre-designed miScript primer catalogue, whereas primers for piRNA and tRF detection were designed using the Qiagen custom miScript primer service (Additional file 7). RNU6B was used as endogenous control. All samples were run in triplicates and the relative expression was calculated using the equation RQ = 2^-ΔΔCT^. CT values >35 were regarded as negative.

## Additional files

Additional file 1:
**Absolute distribution of small RNA sequences in three classes according to annotation.**
Additional file 2:
**tRNA-derived small RNA expression in normal and TGCT samples.**
Additional file 3:
**Correlation between tRFs and tRNA halves in normal and TGCT samples.**
Additional file 4:
**Table indicating differentially expressed miRNAs with** ***p*** **< 10** ^**−6**^ **when adjusting for multiple testing with the Benjamini-Hochberg procedure.**
Additional file 5:
**Volcano plot indicating the relationship between the Log2 fold change and p-values (Benjamini-Hochberg adjusted) for all sequences overlapping with piRNABank.**
Additional file 6:
**Table indicating the top 10 differentially expressed piRNAs when including sequences overlapping with piRNABank only.**
Additional file 7:
**qPCR primer overview.**

## Abbreviations

CIS: Carcinoma *in situ*
EC: Embryonic carcinoma
LATS2: Large Tumor Suppressor homolog 2
miRNA: microRNA
ncRNA: non-coding RNAs
nt: nucleotide
PGCs: primordial germ cells
piRNA: PIWI-interacting RNA
sncRNA: small non-coding RNAs
TGCT: Testicular germ cell tumor
tRFs: tRNA fragments

## References

[1] Bertone P, Stolc V, Royce TE, Rozowsky JS, Urban AE, Zhu X, Rinn JL, Tongprasit W, Samanta M, Weissman S (2004). Global identification of human transcribed sequences with genome tiling arrays. Science.

[2] Esteller M (2011). Non-coding RNAs in human disease. Nat Rev Genet.

[3] Farazi TA, Juranek SA, Tuschl T (2008). The growing catalog of small RNAs and their association with distinct Argonaute/Piwi family members. Development.

[4] Peters L, Meister G (2007). Argonaute proteins: mediators of RNA silencing. Mol Cell.

[5] Girard A, Sachidanandam R, Hannon GJ, Carmell MA (2006). A germline-specific class of small RNAs binds mammalian Piwi proteins. Nature.

[6] Deng W, Lin HF (2002). Miwi, a murine homolog of piwi, encodes a cytoplasmic protein essential for spermatogenesis. Dev Cell.

[7] Hutvagner G, Simard MJ (2008). Argonaute proteins: key players in RNA silencing. Nat Rev Mol Cell Biol.

[8] Calin GA, Ferracin M, Cimmino A, Di Leva G, Shimizu M, Wojcik SE, Iorio MV, Visone R, Sever NI, Fabbri M (2005). A MicroRNA signature associated with prognosis and progression in chronic lymphocytic leukemia. N Engl J Med.

[9] Lu J, Getz G, Miska EA, Alvarez-Saavedra E, Lamb J, Peck D, Sweet-Cordero A, Ebert BL, Mak RH, Ferrando AA (2005). MicroRNA expression profiles classify human cancers. Nature.

[10] Wu Q, Song R, Ortogero N, Zheng H, Evanoff R, Small CL, Griswold MD, Namekawa SH, Royo H, Turner JM (2012). The RNase III enzyme DROSHA is essential for microRNA production and spermatogenesis. J Biol Chem.

[11] Smorag L, Zheng Y, Nolte J, Zechner U, Engel W, Pantakani DV (2012). MicroRNA signature in various cell types of mouse spermatogenesis: evidence for stage-specifically expressed miRNA-221, −203 and -34b-5p mediated spermatogenesis regulation. Biol Cell.

[12] Gillis AJM, Stoop HJ, Hersmus R, Oosterhuis JW, Sun Y, Chen C, Guenther S, Sherlock J, Veltman I, Baeten J (2007). High-throughput microRNAome analysis in human germ cell tumours. J Pathol.

[13] Voorhoeve PM, le Sage C, Schrier M, Gillis AJM, Stoop H, Nagel R, Liu YP, van Duijse J, Drost J, Griekspoor A (2006). A genetic screen implicates miRNA-372 and miRNA-373 as oncogenes in testicular germ cell tumors. Cell.

[14] Huszar JM, Payne CJ (2013). MicroRNA 146 (Mir146) Modulates Spermatogonial Differentiation by Retinoic Acid in Mice. Biol Reprod.

[15] Aravin A, Gaidatzis D, Pfeffer S, Lagos-Quintana M, Landgraf P, Iovino N, Morris P, Brownstein MJ, Kuramochi-Miyagawa S, Nakano T (2006). A novel class of small RNAs bind to MILI protein in mouse testes. Nature.

[16] Lau NC, Seto AG, Kim J, Kuramochi-Miyagawa S, Nakano T, Bartel DP, Kingston RE (2006). Characterization of the piRNA complex from rat testes. Science.

[17] Grivna ST, Beyret E, Wang Z, Lin H (2006). A novel class of small RNAs in mouse spermatogenic cells. Genes Dev.

[18] Cheng J, Guo JM, Xiao BX, Miao Y, Jiang Z, Zhou H, Li QN (2011). piRNA, the new non-coding RNA, is aberrantly expressed in human cancer cells. Clin Chim Acta.

[19] Lu Y, Li C, Zhang K, Sun H, Tao D, Liu Y, Zhang S, Ma Y (2010). Identification of piRNAs in Hela cells by massive parallel sequencing. BMB Rep.

[20] Keam SP, Young PE, McCorkindale AL, Dang TH, Clancy JL, Humphreys DT, Preiss T, Hutvagner G, Martin DI, Cropley JE (2014). The human Piwi protein Hiwi2 associates with tRNA-derived piRNAs in somatic cells. Nucleic Acids Res.

[21] Yan Z, Hu HY, Jiang X, Maierhofer V, Neb E, He L, Hu Y, Hu H, Li N, Chen W (2011). Widespread expression of piRNA-like molecules in somatic tissues. Nucleic Acids Res.

[22] Ishizu H, Siomi H, Siomi MC (2012). Biology of PIWI-interacting RNAs: new insights into biogenesis and function inside and outside of germlines. Genes Dev.

[23] Aravin AA, Sachidanandam R, Girard A, Fejes-Toth K, Hannon GJ (2007). Developmentally regulated piRNA clusters implicate MILI in transposon control. Science.

[24] Carmell MA, Girard A, van de Kant HJG, Bourc'his D, Bestor TH, de Rooij DG, Hannon GJ (2007). MIWI2 is essential for spermatogenesis and repression of transposons in the mouse male germline. Dev Cell.

[25] Brennecke J, Aravin AA, Stark A, Dus M, Kellis M, Sachidanandam R, Hannon GJ (2007). Discrete small RNA-generating loci as master regulators of transposon activity in Drosophila. Cell.

[26] Cox DN, Chao A, Baker J, Chang L, Qiao D, Lin H (1998). A novel class of evolutionarily conserved genes defined by piwi are essential for stem cell self-renewal. Genes Dev.

[27] Aravin AA, Sachidanandam R, Bourc'his D, Schaefer C, Pezic D, Toth KF, Bestor T, Hannon GJ (2008). A piRNA pathway primed by individual transposons is linked to de novo DNA methylation in mice. Mol Cell.

[28] Kuramochi-Miyagawa S, Kimura T, Ijiri TW, Isobe T, Asada N, Fujita Y, Ikawa M, Iwai N, Okabe M, Deng W (2004). Mili, a mammalian member of piwi family gene, is essential for spermatogenesis. Development.

[29] Thomson T, Lin H (2009). The biogenesis and function of PIWI proteins and piRNAs: progress and prospect. Annu Review Cell Dev Biol.

[30] Le Thomas A, Toth KF, Aravin AA (2014). To be or not to be a piRNA: genomic origin and processing of piRNAs. Genome Biol.

[31] Sobala A, Hutvagner G (2011). Transfer RNA-derived fragments: origins, processing, and functions. Wiley Interdis Rev RNA.

[32] Thompson DM, Lu C, Green PJ, Parker R (2008). tRNA cleavage is a conserved response to oxidative stress in eukaryotes. RNA.

[33] Yamasaki S, Ivanov P, Hu GF, Anderson P (2009). Angiogenin cleaves tRNA and promotes stress-induced translational repression. J Cell Biol.

[34] Ivanov P, Emara MM, Villen J, Gygi SP, Anderson P (2011). Angiogenin-induced tRNA fragments inhibit translation initiation. Mol Cell.

[35] Peng H, Shi J, Zhang Y, Zhang H, Liao S, Li W, Lei L, Han C, Ning L, Cao Y (2012). A novel class of tRNA-derived small RNAs extremely enriched in mature mouse sperm. Cell Res.

[36] Skakkebaek NE, Berthelsen JG, Giwercman A, Muller J (1987). Carcinoma-in-situ of the testis: possible origin from gonocytes and precursor of all types of germ cell tumours except spermatocytoma. Int J Androl.

[37] Chia VM, Quraishi SM, Devesa SS, Purdue MP, Cook MB, McGlynn KA (2010). International Trends in the Incidence of Testicular Cancer, 1973–2002. Cancer Epidem Biomar.

[38] McGlynn KA, Devesa SS, Sigurdson AJ, Brown LM, Tsao L, Tarone RE (2003). Trends in the incidence of testicular germ cell tumors in the United States. Cancer.

[39] Sonke GS, Chang S, Strom SS, Sweeney AM, Annegers JF, Sigurdson AJ (2007). Prenatal and perinatal risk factors and testicular cancer: a hospital-based case–control study. Oncology Res.

[40] Bernstein L, Depue RH, Ross RK, Judd HL, Pike MC, Henderson BE (1986). Higher maternal levels of free estradiol in first compared to second pregnancy: early gestational differences. J Natl Cancer Inst.

[41] Aschim EL, Grotmol T, Tretli S, Haugen TB (2005). Is there an association between maternal weight and the risk of testicular cancer? An epidemiologic study of Norwegian data with emphasis on World War II. Int J Cancer.

[42] Kristensen DG, Nielsen JE, Jorgensen A, Skakkebaek NE, Rajpert-De Meyts E, Almstrup K (2013). Evidence that active demethylation mechanisms maintain the genome of carcinoma in situ cells hypomethylated in the adult testis. Br J Cancer.

[43] Ferreira HJ, Heyn H, GarciaDel Muro X, Vidal A, Larriba S, Munoz C, Villanueva A, Esteller M (2014). Epigenetic loss of the PIWI/piRNA machinery in human testicular tumorigenesis. Epigenetics.

[44] Lee Y, Ahn C, Han J, Choi H, Kim J, Yim J, Lee J, Provost P, Radmark O, Kim S (2003). The nuclear RNase III Drosha initiates microRNA processing. Nature.

[45] Luteijn MJ, Ketting RF (2013). PIWI-interacting RNAs: from generation to transgenerational epigenetics. Nature Rev Gen.

[46] Sai Lakshmi S, Agrawal S (2008). piRNABank: a web resource on classified and clustered Piwi-interacting RNAs. Nucleic Acids Res.

[47] Martens-Uzunova ES, Olvedy M, Jenster G (2013). Beyond microRNA--novel RNAs derived from small non-coding RNA and their implication in cancer. Cancer Lett.

[48] Gan H, Lin X, Zhang Z, Zhang W, Liao S, Wang L, Han C (2011). piRNA profiling during specific stages of mouse spermatogenesis. RNA.

[49] Beyret E, Liu N, Lin H (2012). piRNA biogenesis during adult spermatogenesis in mice is independent of the ping-pong mechanism. Cell Res.

[50] Ha H, Song J, Wang S, Kapusta A, Feschotte C, Chen KC, Xing J (2014). A comprehensive analysis of piRNAs from adult human testis and their relationship with genes and mobile elements. BMC Genomics.

[51] Almstrup K, Leffers H, Lothe RA, Skakkebaek NE, Sonne SB, Nielsen JE, Rajpert-De Meyts E, Skotheim RI (2007). Improved gene expression signature of testicular carcinoma in situ. Int J Androl.

[52] Cole C, Sobala A, Lu C, Thatcher SR, Bowman A, Brown JW, Green PJ, Barton GJ, Hutvagner G (2009). Filtering of deep sequencing data reveals the existence of abundant Dicer-dependent small RNAs derived from tRNAs. RNA.

[53] Burroughs AM, Ando Y, de Hoon MJ, Tomaru Y, Suzuki H, Hayashizaki Y, Daub CO (2011). Deep-sequencing of human Argonaute-associated small RNAs provides insight into miRNA sorting and reveals Argonaute association with RNA fragments of diverse origin. RNA Biol.

[54] Sobala A, Hutvagner G (2013). Small RNAs derived from the 5' end of tRNA can inhibit protein translation in human cells. RNA Biol.

[55] Pavon-Eternod M, Gomes S, Geslain R, Dai Q, Rosner MR, Pan T (2009). tRNA over-expression in breast cancer and functional consequences. Nucleic Acids Res.

[56] Ushida H, Kawakami T, Minami K, Chano T, Okabe H, Okada Y, Okamoto K (2012). Methylation profile of DNA repetitive elements in human testicular germ cell tumor. Molecular Carcinog.

[57] Murray MJ, Saini HK, van Dongen S, Palmer RD, Muralidhar B, Pett MR, Piipari M, Thornton CM, Nicholson JC, Enright AJ (2010). The two most common histological subtypes of malignant germ cell tumour are distinguished by global microRNA profiles, associated with differential transcription factor expression. Mol Cancer.

[58] Palmer RD, Murray MJ, Saini HK, van Dongen S, Abreu-Goodger C, Muralidhar B, Pett MR, Thornton CM, Nicholson JC, Enright AJ (2010). Malignant germ cell tumors display common microRNA profiles resulting in global changes in expression of messenger RNA targets. Cancer Res.

[59] Lin SL, Chang DC, Chang-Lin S, Lin CH, Wu DT, Chen DT, Ying SY (2008). Mir-302 reprograms human skin cancer cells into a pluripotent ES-cell-like state. RNA (New York, NY).

[60] Liu L, Lian J, Zhang H, Tian H, Liang M, Yin M, Sun F (2013). MicroRNA-302a sensitizes testicular embryonal carcinoma cells to cisplatin-induced cell death. J Cell Physiol.

[61] Gregory PA, Bert AG, Paterson EL, Barry SC, Tsykin A, Farshid G, Vadas MA, Khew-Goodall Y, Goodall GJ (2008). The miR-200 family and miR-205 regulate epithelial to mesenchymal transition by targeting ZEB1 and SIP1. Nat Cell Biol.

[62] Neves R, Scheel C, Weinhold S, Honisch E, Iwaniuk KM, Trompeter HI, Niederacher D, Wernet P, Santourlidis S, Uhrberg M (2010). Role of DNA methylation in miR-200c/141 cluster silencing in invasive breast cancer cells. BMC Res Notes.

[63] Hu M, Xia M, Chen X, Lin Z, Xu Y, Ma Y, Su L (2010). MicroRNA-141 regulates Smad interacting protein 1 (SIP1) and inhibits migration and invasion of colorectal cancer cells. Dig Dis Sci.

[64] Snowdon J, Zhang X, Childs T, Tron VA, Feilotter H (2011). The microRNA-200 family is upregulated in endometrial carcinoma. PLoS One.

[65] Iorio MV, Visone R, Di Leva G, Donati V, Petrocca F, Casalini P, Taccioli C, Volinia S, Liu CG, Alder H (2007). MicroRNA signatures in human ovarian cancer. Cancer Res.

[66] McDonald JS, Milosevic D, Reddi HV, Grebe SK, Algeciras-Schimnich A (2011). Analysis of circulating microRNA: preanalytical and analytical challenges. Clin Chem.

[67] Bezan A, Gerger A, Pichler M (2014). MicroRNAs in testicular cancer: implications for pathogenesis, diagnosis, prognosis and therapy. Anticancer Res.

[68] Dieckmann KP, Spiekermann M, Balks T, Flor I, Loning T, Bullerdiek J, Belge G (2012). MicroRNAs miR-371-3 in serum as diagnostic tools in the management of testicular germ cell tumours. Br J Cancer.

[69] Spiekermann M, Belge G, Winter N, Ikogho R, Balks T, Bullerdiek J, Dieckmann KP (2015). MicroRNA miR-371a-3p in serum of patients with germ cell tumours: evaluations for establishing a serum biomarker. Andrology.

[70] Belge G, Dieckmann KP, Spiekermann M, Balks T, Bullerdiek J (2012). Serum levels of microRNAs miR-371-3: a novel class of serum biomarkers for testicular germ cell tumors?. Eur Urol.

[71] Syring I, Bartels J, Holdenrieder S, Kristiansen G, Muller SC, Ellinger J (2015). Circulating Serum miRNA (miR-367-3p, miR-371a-3p, miR-372-3p and miR-373-3p) as Biomarkers in Patients with Testicular Germ Cell Cancer. J Urol.

[72] Skotheim RI, Lind GE, Monni O, Nesland JM, Abeler VM, Fossa SD, Duale N, Brunborg G, Kallioniemi I, Andrews PW (2005). Differentiation of human embryonal carcinomas in vitro and in vivo reveals expression profiles relevant to normal development. Cancer Res.

[73] Crooks GE, Hon G, Chandonia JM, Brenner SE (2004). WebLogo: a sequence logo generator. Genome Res.

[74] Griffiths-Jones S, Grocock RJ, van Dongen S, Bateman A, Enright AJ (2006). miRBase: microRNA sequences, targets and gene nomenclature. Nucleic Acids Res.

[75] Quinlan AR, Hall IM (2010). BEDTools: a flexible suite of utilities for comparing genomic features. Bioinformatics.

[76] Liao Y, Smyth GK, Shi W (2014). FeatureCounts: an efficient general purpose program for assigning sequence reads to genomic features. Bioinformatics.

[77] Anders S, Huber W (2010). Differential expression analysis for sequence count data. Genome Biol.

