# Supplementary material for: Profiling of the small RNA populations in human testicular germ cell tumors shows global loss of piRNAs
Source: Mol Cancer. 2015 Aug 12;14:153. doi: 10.1186/s12943-015-0411-4 (PMC4533958; doi:10.1186/s12943-015-0411-4)
Supplement: Additional file 1: — Absolute distribution of small RNA sequences in three classes according to annotation. [file 12943_2015_411_MOESM1_ESM.pdf]

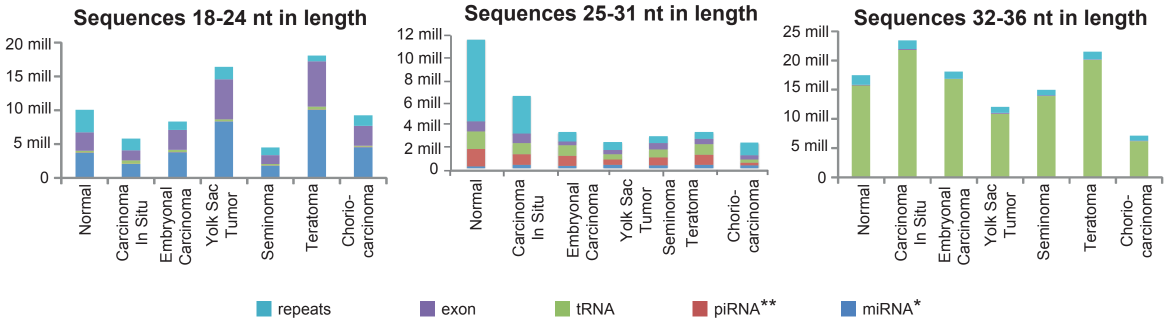

Absolute distribution of small RNA sequences in three classes according to annotation.
The sequences are divided into three categories based on the peaks in the size distribution, corresponding to miRNAs, piRNAs and longer sequences of previously unknown origin. The histograms indicate the average sequence count per sample in each group.
* As annotated by miRBase, ** As annotated by piRNABank
