# Supplementary material for: Profiling of the small RNA populations in human testicular germ cell tumors shows global loss of piRNAs
Source: Mol Cancer. 2015 Aug 12;14:153. doi: 10.1186/s12943-015-0411-4 (PMC4533958; doi:10.1186/s12943-015-0411-4)
Supplement: Additional file 2: — tRNA-derived small RNA expression in normal and TGCT samples. [file 12943_2015_411_MOESM2_ESM.pdf]

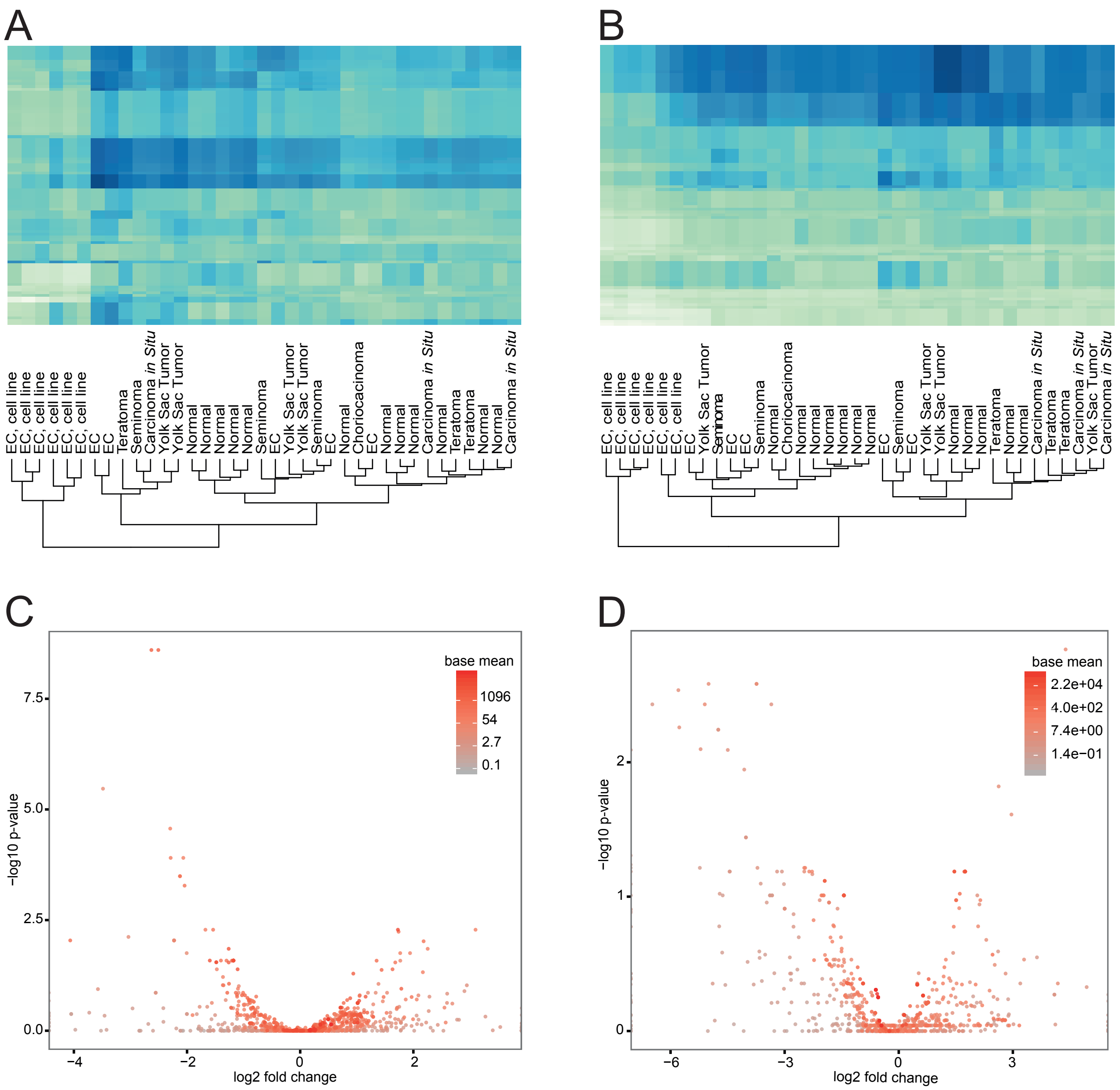


**tRNA-derived small RNA expression in normal and TGCT samples.**
**A** Heatmap showing the expression data of the most highly expressed tRFs (variance stabilization transformed data). The dendrogram (bottom) indicates clustering according to expression profiles. **B** Heatmap showing the expression data of the most highly expressed tRNA halves (variance stabilization transformed data). The dendrogram (bottom) indicates clustering according to expression profiles. **C** Volcano plot indicating the relationship between the Log2 fold change and p-values (Benjamini-Hochberg adjusted) for tRFs when comparing TGCT with normal samples. The CIS samples are included in the TGCT group. Color coding indicates the expression level as a function of normalized counts (BaseMean). **D** Volcano plot indicating the relationship between the Log2 fold change and p-values (Benjamini-Hochberg adjusted) for tRNA halves when comparing TGCT with normal samples. The CIS samples are included in the TGCT group. Color coding indicates the expression level as a function of normalized counts (BaseMean).
