# Supplementary material for: Profiling of the small RNA populations in human testicular germ cell tumors shows global loss of piRNAs
Source: Mol Cancer. 2015 Aug 12;14:153. doi: 10.1186/s12943-015-0411-4 (PMC4533958; doi:10.1186/s12943-015-0411-4)
Supplement: Additional file 3: — Correlation between tRFs and tRNA halves in normal and TGCT samples. [file 12943_2015_411_MOESM3_ESM.pdf]

Additional File 3

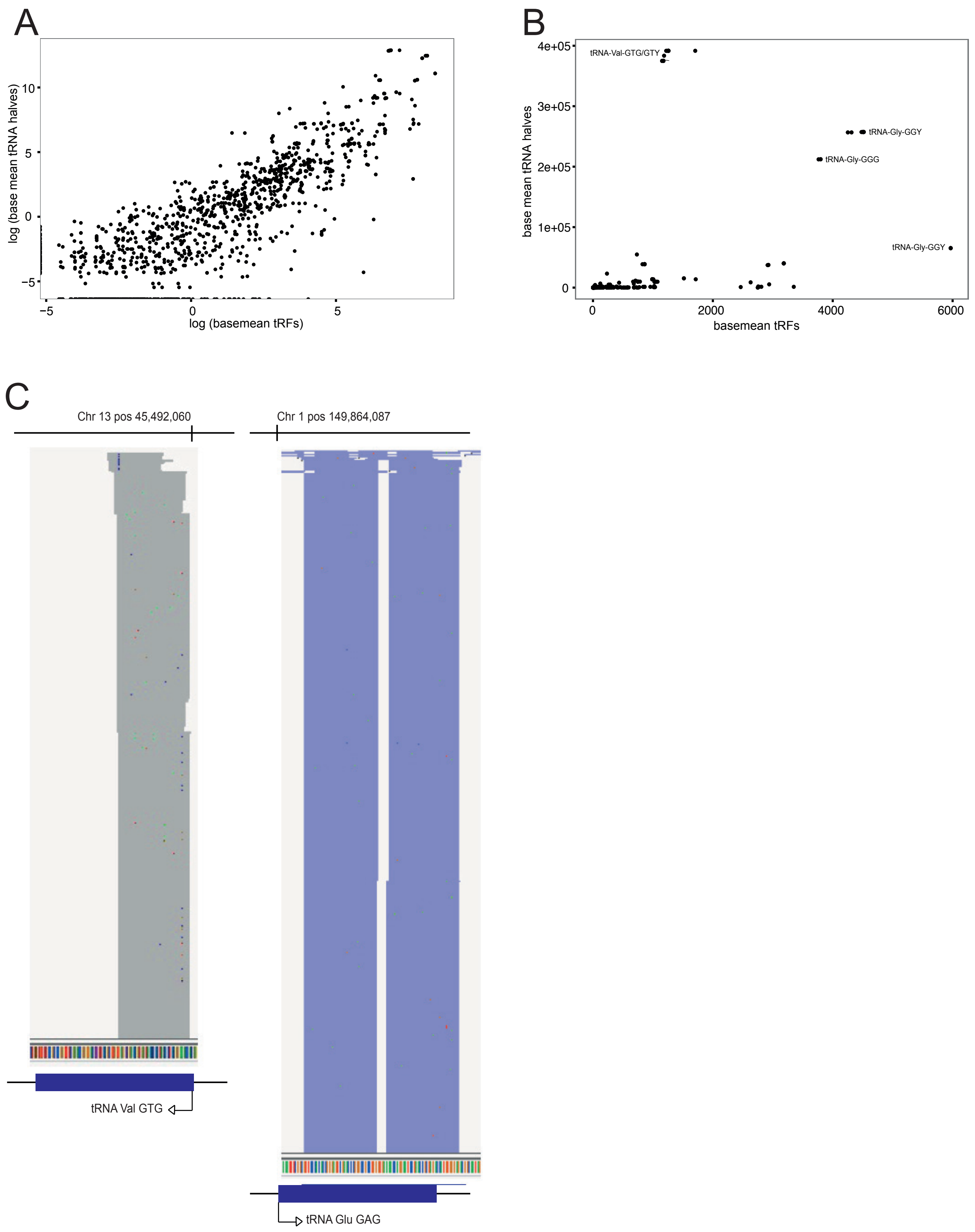

Correlation between tRFs and tRNA halves in normal and TGCT samples. Correlation plots indicating the relationship between tRNA isotypes in the tRF and tRNA halve populations. The plots are prepared using a log scale (**A**) and a normal scale (**B**). **C** Examples of the distribution of tRNA-derived small RNA sequence in two representative tRNA genes. Most tRNA-derived sequences are derived from the 5′ end of mature tRNAs (left), whereas a few tRNAs give rise to both 5′ and 3′ sequences(right). Horizontal grey and blue lines represent sequences derived from tRNAs. Multicolored horizontal bars represent individual bases.
