# Supplementary material for: Profiling of the small RNA populations in human testicular germ cell tumors shows global loss of piRNAs
Source: Mol Cancer. 2015 Aug 12;14:153. doi: 10.1186/s12943-015-0411-4 (PMC4533958; doi:10.1186/s12943-015-0411-4)
Supplement: Additional file 4: — Table indicating differentially expressed miRNAs with p < 10 −6 when adjusting for multiple testing with the Benjamini-Hochberg procedure. [file 12943_2015_411_MOESM4_ESM.pdf]

Differentially expressed miRNAs with p<10-6 when adjusting for multiple testing with the Benjamini-Hochberg procedure.

	miRNA	Log2 Fold Change	p-value (adjusted)
1	hsa-miR-302d-3p	11,25	1,95E-13
2	hsa-miR-302a-3p	11,65	1,23E-12
3	hsa-mir-302b	11,38	1,23E-12
4	hsa-mir-302a	11,18	7,26E-12
5	hsa-miR-367-3p	10,20	1,48E-11
6	hsa-miR-371a-5p	9,29	2,09E-11
7	hsa-miR-371b-3p	9,29	2,09E-11
8	hsa-miR-302c-5p	Inf	3,45E-11
9	hsa-miR-302d-5p	Inf	1,38E-10
10	hsa-miR-302c-3p	11,32	1,38E-10
11	hsa-miR-371a-3p	8,86	2,28E-09
12	hsa-miR-371b-5p	8,86	2,28E-09
13	hsa-miR-372-3p	8,35	5,59E-09
14	hsa-miR-373-3p	8,10	5,37E-09
15	hsa-miR-205-5p	8,43	5,59E-08
16	hsa-miR-302b-5p	10,71	5,59E-08
17	hsa-miR-302e	Inf	5,26E-07
18	hsa-miR-372-5p	7,67	5,26E-07
19	hsa-miR-141-3p	7,15	1,14E-06
20	hsa-miR-200c-3p	8,19	1,40E-06
21	hsa-miR-141-5p	8,69	1,91E-06
22	hsa-miR-373-5p	6,26	2,66E-06
23	hsa-miR-7159-3p	-5,68	6,52E-06
