# Supplementary material for: Profiling of the small RNA populations in human testicular germ cell tumors shows global loss of piRNAs
Source: Mol Cancer. 2015 Aug 12;14:153. doi: 10.1186/s12943-015-0411-4 (PMC4533958; doi:10.1186/s12943-015-0411-4)
Supplement: Additional file 5: — Volcano plot indicating the relationship between the Log2 fold change and p-values (Benjamini-Hochberg adjusted) for all sequences overlapping with piRNABank. [file 12943_2015_411_MOESM5_ESM.pdf]

Additional File 5

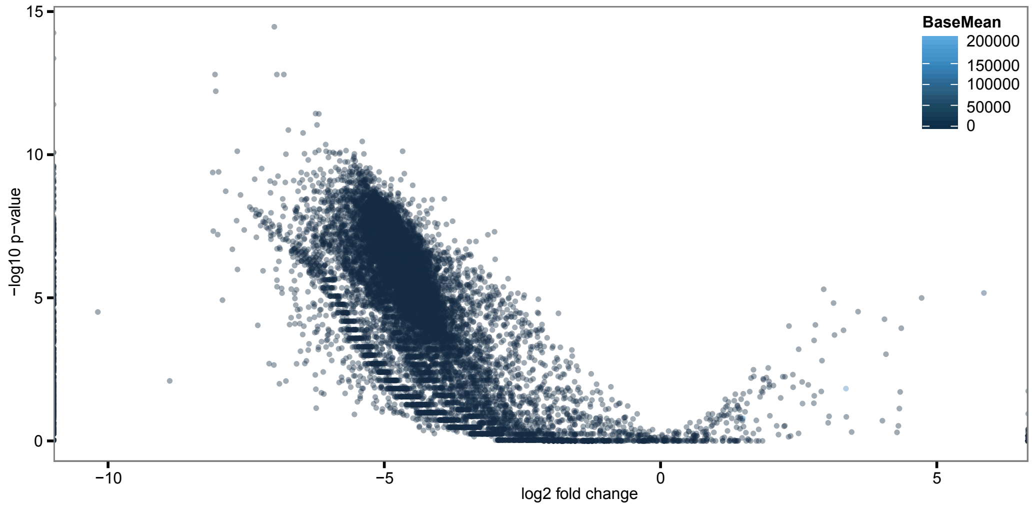

Expression of piRNABank sequences. Volcano plot indicating the relationship between the Log2 fold change and p-values (Benjamini-Hochberg adjusted) for sequences overlapping with piRNABank when comparing TGCT with normal samples. The CIS samples are included in the TGCT group. Color coding indicates the expression level as a function of normalized counts (BaseMean).
