# Supplementary material for: Profiling of the small RNA populations in human testicular germ cell tumors shows global loss of piRNAs
Source: Mol Cancer. 2015 Aug 12;14:153. doi: 10.1186/s12943-015-0411-4 (PMC4533958; doi:10.1186/s12943-015-0411-4)
Supplement: Additional file 6: — Table indicating the top 10 differentially expressed piRNAs when including sequences overlapping with piRNABank only. [file 12943_2015_411_MOESM6_ESM.pdf]

Top 10 differentially expressed piRNAs overlapping with sequences from piRNABank (Benjamini-Hochberg adjusted)

	id	log2FoldChange	p adjusted
1	hsa_piR_008857	-6.991032	3.407340e-15
2	hsa_piR_003068	-Inf	5.542997e-15
3	hsa_piR_014984	-Inf	4.332005e-14
4	hsa_piR_004874	-6.818212	1.583720e-13
5	hsa_piR_002915	-6.944997	1.583720e-13
6	hsa_piR_003656	-8.064095	1.583720e-13
7	hsa_piR_012719	-8.050329	6.081981e-13
8	hsa_piR_004362	-Inf	1.766860e-12
9	hsa_piR_004186	-6.243533	3.672874e-12
10	hsa_piR_015508	-6.184461	3.753682e-12
