# Supplementary material for: Profiling of the small RNA populations in human testicular germ cell tumors shows global loss of piRNAs
Source: Mol Cancer. 2015 Aug 12;14:153. doi: 10.1186/s12943-015-0411-4 (PMC4533958; doi:10.1186/s12943-015-0411-4)
Supplement: Additional file 7: — qPCR primer overview. [file 12943_2015_411_MOESM7_ESM.pdf]

qPCR primers for small RNA expression validation

Small RNA name*	Assay type (Qiagen)	Sequence** / order number
RNU6B	predesigned miScript Primer Assay	MS00033740
miR-302b-3p	predesigned miScript Primer Assay	MS00003906
miR-372-3p	predesigned miScript Primer Assay	MS00004067
miR-373-3p	predesigned miScript Primer Assay	MS00031815
piR-006113	custom miScript Primer Assay	UUGGGAAAUGCAACAUUUGGGCAGGA
piR-019085	custom miScript Primer Assay	ACAAGACAAAGAGAUAAAGAGAAAGCA
piR-007509	custom miScript Primer Assay	AGAAUUUAUGGUUUGUAGGGCAUGA
tRF Glu-GAG, chr13	custom miScript Primer Assay	UCCCACAUGGUCUAGCG
tRF Glu-GAG, chr15	custom miScript Primer Assay	UCCCACAUGGUCUAGCGGU
tRF Gly-GGA, chr20	custom miScript Primer Assay	GCGUUGGUGGUAUAGUGG

* The piRNA (piR) and tRF sequences were found using IGV visualization of sequence reads
** The sequence used in the Qiagen miScript service for custom miRNA primer design
